# IFT and total mutational load

**DOI:** 10.1186/2046-2530-1-S1-O24

**Published:** 2012-11-16

**Authors:** N Katsanis

**Affiliations:** 1 Center for Human Disease Modeling, Department of Cell Biology, Duke University, USA

## 

Defects of the primary cilium and its anchoring structure, the basal body, cause a number of human genetic disorders, collectively termed ciliopathies, since they are characterized by an overlapping range of phenotypes that include retinal degeneration, polydactyly, renal and hepatic fibrosis, obesity and a complex range of cognitive and neurodevelopmental defects. Recent data have also shown that some ciliopathies overlap not only phenotypically, but also genetically by contributing epistatic alleles that can modulate the phenotypic expressivity and penetrance. As such, the primary cilium and its associated signaling represent a useful model to understand the mechanism of total mutational load in a biological system. Towards that end, we have initiated systematic sequencing, copy number variation analysis and functional evaluation of genetic lesions of ciliary genes in a range of ciliopathy phenotypes and, using a large allelic series, have constructed models of epistasis in oligogenic disorders that suggest an intricate interaction between rare and common alleles to modulate penetrance and expressivity. Such studies will ultimately empower the predictive nature of the genotype and inform clinical management and treatment. Importantly, coupling deep medical resequencing with systematic in vivo and in vitro functional testing offers significantly improved resolution and increased appreciation of the complexity and architecture of genetic disease.

